# Evolution of some liver function markers after treatment in patients with schizophrenia and bipolar disorder

**DOI:** 10.1192/j.eurpsy.2023.1951

**Published:** 2023-07-19

**Authors:** S. Sellami, M. Maalej, M. Ayadi, M. Naifar, M. Maalej, F. Ayadi

**Affiliations:** 1 Psychiatry “C” department, Hedi Chaker University Hospital; 2 PsycLaboratory of Research “Molecular Basis of Human Diseases”, LR19ES13, Faculty of medecine of Sfax; 3 Psychiatry “C” department, Hedi Chaker University Hospital; 4 Laboratory of Biochemistry, Faculty of Medicine of Sfax & Habib Bourguiba Hospital; 5 Laboratory of Biochemistry, Faculty of Medicine of Sfax & Habib Bourguiba Hospital, Sfax, Tunisia

## Abstract

**Introduction:**

The prevalence of alterations of liver function tests in patients with schizophrenia and bipolar disorders is not well known. These alterations are often considered as side effects of medication

**Objectives:**

Our study aimed to evaluate and compare liver function before and after treatment in patients with schizophrenia (SCZ), schizo-affective disorder (SCA) and bipolar disorder (BD).

**Methods:**

This was a prospective study among patients with SCZ, SCA and BD according to DSM-5 criteria. Patients, from the “C” psychiatry department of Hedi Chaker University Hospital in Sfax, were assessed during both acute and remission phases in their illness. The acute phase (T0) assessment was made in drug-free patients from june 2016 to july 2018. As for the remission phase (T1), it was made between november 2019 and march 2020. Blood tests were performed in the Laboratory of Biochemistry at Habib Bourguiba University Hospital in Sfax. Clinical and biological parameters of patients were compared with those of healthy controls. Biological assessment consisted mainly in Aspartate Aminotransferase (AST), Alanine Aminotransferase (ALT) and Albumine.

**Results:**

Thirty patients were included in our study. Their mean age was 35.83 ± 12.24 years and they were all males. They suffered from SCZ in 33.33% of cases, from SCA in 26.66% of cases and from BD in 40% of cases. Psychoactive substance use was common among 80% of patients. In the remission phase, 90% were polymedicated with use of antipsychotics in 83% of cases and mood stabilisers in 53% of cases. Table 1 shows the evolution of the studied liver function markers in our patients.

**Table 1:** evolution of some liver function markers in patients

***Markers***		***T0***	***T1***	***p***
**AST (UI/L)**	**Patients**	33,22 ± 23,18	19,34 ± 4,97	**<0,001**
	**Controls**	22,27±6,91	**<0,05**^**e,b**^	
**ALT (UI/L)**	**Patients**	19,59 ± 13,2	13,17 ± 11,39	**0,003**
	**Controls**	20,63±11,08	**<0,05**^**e,a,b**^	
**Albumine (g/l)**	**Patients**	42,35±4,86	47,79±3,18	**<0,001**
	**Controls**	46,19±3,95	>0,05	

^a^: significant difference between patients with SCZ (T1) and controls; b: significant difference between patients with BD (T1) and controls; e: significant difference between patients (T1) and controls

**Conclusions:**

Our results showed an improvement of liver function in patients with SCZ and BD after treatment. This suggests that liver function alterations are due to these diseases rather than the medication.

**Disclosure of Interest:**

None Declared

